# Interplay of mental state, personality, and popularity among peers in shaping belongingness of first-year students: A cross-sectional study

**DOI:** 10.1371/journal.pmen.0000019

**Published:** 2024-07-31

**Authors:** Audrey Zhang, Fjorda Kazazi, Kevin Tang, Peter Howell

**Affiliations:** 1 Department of Experimental Psychology, University College London, London, United Kingdom; 2 Department of English Language and Linguistics, Heinrich Heine University Düsseldorf, Düsseldorf, Germany; University of Turin, ITALY

## Abstract

Belonging to a university shapes wellbeing and academic outcomes for first-year students, yet this belongingness is harder to achieve for those from lower socio-economic backgrounds. This study delved into the flexible construct of status—the individual’s perceived position within the university’s social hierarchy and the strategy they adopt to achieve that position—and its impact on their belongingness. The objective was to identify key psychological contributors that could impact first-year Psychology students expected social status and thereby their belongingness. A cross-sectional study tested first year Psychology students entering university in 2021 and 2022.The first-year students completed a battery of questionnaires to ascertain their status, belongingness to the university, mental state, and personalities. Structural equation modelling (SEM) was employed to evaluate a social ecological model focusing on belongingness. This analysis investigated the mediating role of peer status (popularity among peers) in the relationship between mental state and belongingness, and the moderating influence of personality traits on the connections between mental state and peer status. Both the mediation and moderation effects were statistically significant after adjusting for gender and ethnicity. The findings offer insights into how university administrations can effectively support students, particularly those from lower socio-economic backgrounds, in enhancing their social status among peers and fostering a stronger belongingness, thereby promoting their overall mental wellbeing and success in their academic pursuits.

## Introduction

Entering university can be daunting. First-year undergraduate students are challenged academically and emotionally: Consequently, most students who drop out of university do so during or immediately after their first year [1, 2]. The transitional experience during the first year is also often accompanied with existential (e.g., control over life), friendship (e.g., time spent with friends), and romantic (e.g., time spent with a romantic partner) loss, as indicated by exploratory factor analysis [3].

Belongingness to the university can be a protective factor during this turbulent period [4]. In the present study, belongingness or belongingness refers to the extent to which a student feels “part of the campus community” or a “member of the campus community”, and also gives them a “belongingness to the campus community” [5]. A belongingness is one of the most frequently cited factors that contributes to first-year students’ academic success and emotional wellbeing [6, 7].

Establishing a belongingness in a university setting is disproportionately challenging for students from lower socioeconomic backgrounds, a difficulty rooted in fixed and systemic socio-economic hierarchies [8, 9]. The university landscape, steeped in middle-class norms, often inadvertently perpetuates a sense of exclusion for those from less affluent backgrounds, creating an environment where their higher socioeconomic counterparts are predisposed to thrive [10, 11]. Within this context, perceived status—defined in this study as an individual’s self-assessed position and the strategic behaviour they engage in within the university’s social hierarchy—emerges as a mutable and influential construct. Unlike the more static determinant of socioeconomic background, expected social status is a lever for enhancing belongingness [12]. Ostrove and Long [13] underscored this distinction by demonstrating the impactful role of expected social status as a predictor of belonging, thus directing attention to its potential as a target for intervention. By focusing on malleable aspects of the student experience, such as expected social status, this research seeks to uncover contributory factors and practical interventions that can foster a heightened belongingness for first-year students, irrespective of their unchangeable socioeconomic origins. In the context of this study, ‘expected social status’ will hereafter be referred to as simply ‘status’.

Despite status being a primary predictor for student belongingness, few university belongingness interventions have been targeted on enhancing student status. Psychological competency, such as mental state like temporal stress and wellness, or personality traits like extraversion and openness could serve as powerful targets for new undergraduates, allowing them to establish popularity and higher social status in campus environment [14–16]. Interventions that targeted mental state, such as an online forum could enhance individuals’ perceived status and belongingness to the user community, but to date these mainly address cancer care and adolescent wellbeing areas [17, 18]. On the other hand, plastic personalities underlie an individual’s tendency to actively engage with novelty, change, and exploration both in the social realm (extraversion) and in the realm of ideas and experiences (openness) [16]. This tendency strengthens the effect of mental state, allowing individuals to achieve higher status faster, and maintain the status for longer [15, 19]. The recent rise of social emotional competency has drawn attention to childhood and adolescent psychological targets for predicting adulthood success, however no study to date has investigated university students’ psychological targets and how they enable a smooth transitioning from home to university.

The purpose of this research, therefore, was to 1. construct a model for predicting the status and belongingness of first-year undergraduate students enrolled in a UK university and 2. test the association of psychological targets, such as mental state and personality traits, on promoting expected social status, thus leading to higher belongingness. Whilst most previous work has treated status and belongingness as mediators to academic achievement [20], motivational outcome, and social-emotional wellbeing [21], little work has focused on understanding the and psychological conditions and traits that might predispose first-year students to higher peer status and belongingness.

## Theoretical framework and models

Existing theoretical models have identified several key factors that influence a student’s belongingness at university [20, 22–25]. For instance, Slaten et al. [20] highlighted the significance of being involved in meaningful group activities, the drive to form relationships, the development of strong friendships, and the impact of a diverse and inclusive university environment. Research also points to the importance of students’ backgrounds, such as belonging to a minority group or coming from families without a history of university attendance [23, 25]. However, most of these models view belongingness from a limited perspective, not fully considering it within a complex social ecology that includes individual, social, and environmental factors [26–28]. Importantly, while studies have consistently shown that peer status is a core predictor of belongingness, few have explored how status functions within an ecological framework. Specifically, there is limited understanding of how status might mediate the effects of other factors on belongingness [29–31].

A trait-state theory suggests that human perceptions of their living environments are shaped by the interplay between transient (state) conditions and enduring (trait) characteristics [32]. For instance, under threatening circumstances, trait neuroticism manifests through various levels of state anxiety, illustrating the interplay between genetic predispositions and environmental stressors [29]. Certain traits are particularly pertinent when encountering new environments, such as entering university for the first time. According to the Unifying Theory of Personality, extraversion and openness are plasticity traits that links to the dopamine system, enhancing motivation and reward-seeking behaviour. These traits are crucial for rapid adaptation to new environments by promoting exploration, creativity, and engagement with novel stimuli. Possessing plasticity traits may also help mitigate the relationship between mental state and status among peers, suggesting that individuals with these traits might experience less impact on their popularity among peers despite temporal mental health challenges. Conversely, stability traits like neuroticism, conscientiousness, and agreeableness, associated with serotonin function, promote long-term consistency and resilience [33]. These traits tend to be crucial for sustaining long-term social stability but less influential in achieving initial peer status [34].

Our study categorizes the influences on students’ belongingness and peer status into two main types of individual-level factors, mental state, and plasticity traits. Mental state factors, encompassing temporal mental wellbeing and stress, are dynamic and heavily influenced by the environment [35–38]. There is a wealth of literature suggesting a sizable impact of mental state on adolescent’s engagement with school. For instance, frequent positive emotions correlate with adaptive coping strategies and higher school engagement. In contrast, prolonged stress is linked to reduced school participation and a diminished sense of belongingness [36, 39, 40]. Meanwhile, university freshmen with more malleable personality traits, or plasticity traits, are more likely to initiate events, quickly form new peer connections, and view the transition to university life as less threatening [41–44]. This underscores the importance of plasticity in fostering initial community belongingness.

### Hypotheses

**Fig 1:**
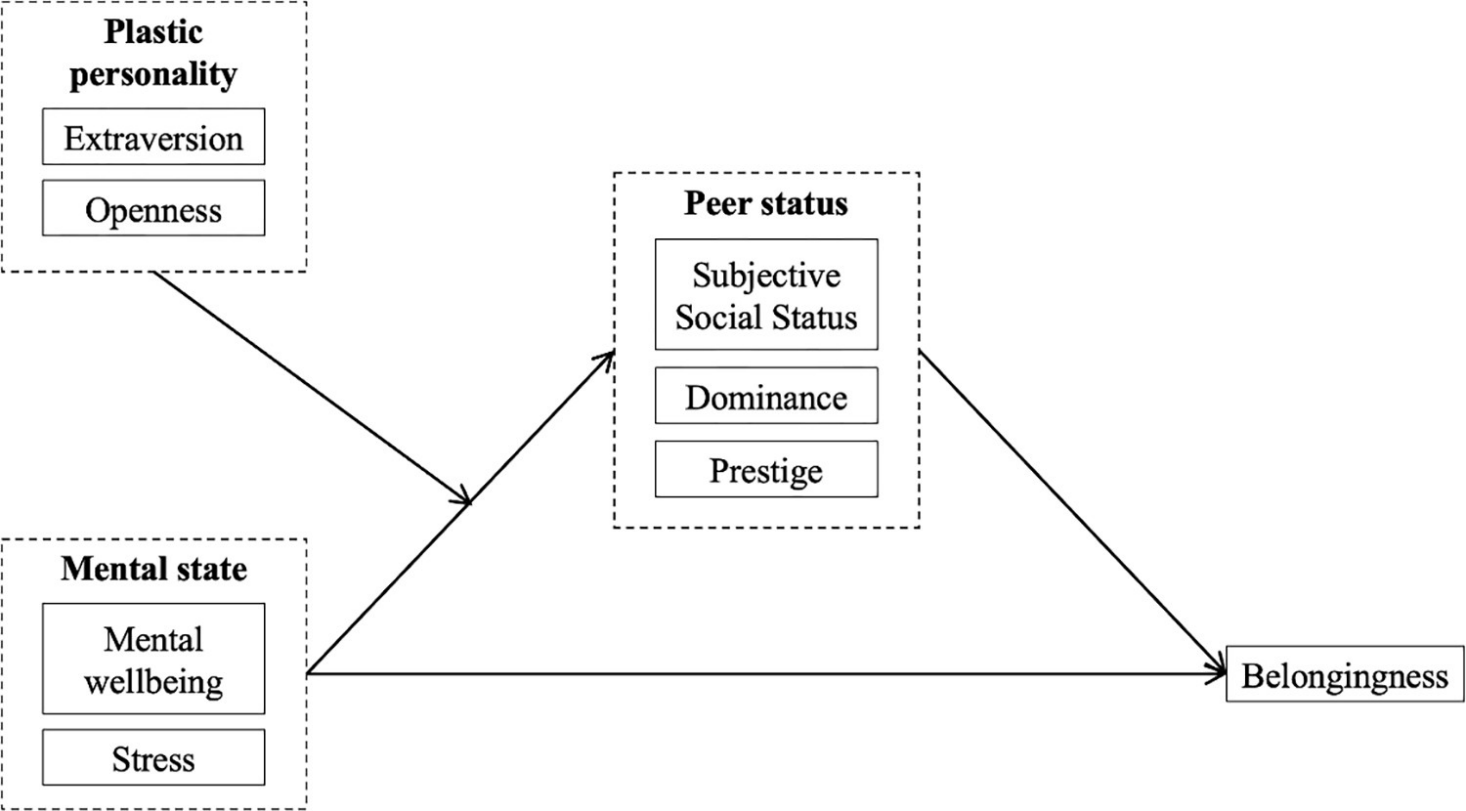
Hypothesized model of status and belongingness of first-year undergraduate students. Dotted-line square represent latent variables.

Based on prior research [45], the conceptual model used in this study assumes that status and belongingness are related to mental state and personality factors (Fig 1). In this study, a battery of self-report questionnaires measuring personality, mental state and dependent variables were distributed to first-year students in a UK university cross-sectionally.

Three hypotheses were formulated:

*Hypothesis 1*: Mental state should be significantly and positively associated with belongingness.
*Hypothesis 2*: Peer status should mediate the relationship between mental state and belongingness.
*Hypothesis 3*: Plasticity traits should moderate the relationship between mental state and belongingness.

## Methods

### Participants

The recruitment period for this study spanned from October 23, 2021, to January 7, 2022. A cohort of 180 first-year BSc Psychology students from University College London (UCL) was randomly sampled and recruited via an online pool system associated with the Department of Psychology and Language Sciences. Participation in this study was part of an educational incentive where participants received one course credit for each of the three test sessions they completed. The number of participants and the demographics for those who completed each test session are detailed in S1 Table.

### Ethics statement

This research was conducted in full compliance with the ethical standards of UCL and was approved by the Department of Psychology and Language Sciences Ethics Committee at UCL.

### Ethical oversight

- Institutional Review Board/Ethics Committee: Department of Psychology and Language Sciences Ethics Committee, UCL.
- Approval Number: Z6364106, dated 2019-10-75, valid until 20/11/2024.

#### Consent procedures

For adult participants: Formal consent was obtained prior to participation in this study. Participants provided written consent, acknowledging their understanding of the research purpose, procedures, and their rights as participants.

For child participants: In alignment with ethical guidelines for research involving minors, written informed consent was obtained from the parents or legal guardians of all child participants under the age of 18. This consent process ensured that guardians were fully informed about the study’s nature, purpose, potential risks, and benefits, and the rights of their children as participants.

This study involved an investigation into the impact of a diversity intervention forum aimed at moderating status and belongingness among first-year undergraduate students at UCL. The consent process emphasized voluntary participation, confidentiality, and the right to withdraw at any time without penalty.

### Apparatus and stimuli

#### The questionnaire

The questionnaires summarized in Table 1 were distributed via Gorilla.sc [46]. Six scales (column three) were employed to measure dependent variable, independent variable, mediator, and moderators. Demographic information was collected.

**Table 1:** Test variables with parameters, questionnaires, and descriptions in three test sessions.

Variables	Parameter	Questionnaires	Description
Dependent variable Mediator	Belongingness Peer status • Subjective Social Status • Dominance and Prestige	Belongingness Scale [47] MacArthur Scale of Subjective Social Status–Youth version [48] Dominance-Prestige Scales [44]	A 30-item, 8-point Likert scale consisted of five subscales on membership, acceptance, affect, desire to fade, and trust. A 10-rung ladder, with the top rung representing students who are most respected in school and the bottom rung representing students who receive the least respect. Participants indicate their perceived position on the ladder. A 17-item, 7-point Likert scale. Eight of the items test for dominance and the rest for prestige.
Independent variable	Mental state • Mental wellbeing	Warwick-Edinburgh mental wellbeing scale [49]	A 14-item, 5-point Likert scale asks participants to rate statements that best describe their feelings and thoughts over the last two weeks.
	• Stress	Perceived Stress Scale [50]	A 10-item, 5-point Likert scale to tap the degree participants found their lives unpredictable, uncontrollable, and overloaded over the last month.
Moderator	Personality	Big Five Personality [51]	A 50-item, 5-point scale with ten items for each trait: extraversion, neuroticism, agreeableness, conscientiousness, and openness to experience.

#### Dependent variable

*Belongingness*. Belongingness was assessed with the adapted Math Belongingness Scale [47]. The adapted scale is an 8-item, 8-point Likert scale. The researchers changed the “math” into “UCL” for some items to fit the purpose of the current experiment. Some example items are “When I am in UCL, I feel a connection with the community” and “When I am in UCL, I feel respected”. This measure assesses students’ feeling of membership and acceptance by their university community.

#### Independent variable

*Mental wellbeing*. Mental wellbeing was tested using the Warwick-Edinburgh mental wellbeing scale (WEMWBS; [49]). The scale is a 14-item scale assessing the positive aspect of mental state, such as participant wellbeing and psychological functioning [52]. In this scale, participants need to rate their experience over the last two weeks on a five-point Likert scale ranging from 1 (‘None of the time’) to 5 (All the time) against the description for each item. An example item is “I’ve been feeling optimistic about the future”. This scale has good internal consistency, test-retest reliability, and construct and criterion validity [52].

*Stress*. Stress was examined using the Perceived Stress Scale (Cronbach’s α > .70, [50]). The scale contains ten items that measure students’ stress levels in the past month. Participants responded to the items on a five-point Likert scale ranging from 1 (‘Never’) to 5 (‘Very often). A sample item is “How often have you been upset because of something that happened unexpectedly?”

#### Mediator

*Peer status*. Three facets were considered to determine peer status: Subjective social status (SSS), dominance, and prestige. Students’ SSS was assessed using the youth version of the MacArthur Scale of SSS [53]. The scale has a single item that asks participants to rank themselves on a 10-point ladder, of which the top (10^th^) rung represent students who were most respected in school and the bottom (1^st^) rung represents those who received the fewest respects. Students ranked themselves relative to other people in their university. SSS captures the degree of social stratification among young adults and reflects their popularity and global self-esteem [53]. Meta-analysis has revealed that high SSS is associated with better mental state [48].

Dominance is the use of force and intimidation to induce fear, and prestige is the sharing of expertise or know-how to gain respect [54]. Both dominance and prestige are pathways to attaining social status [55]. Individuals scoring high in either of these pathways have greater capacities for social influence over others [56]. High dominance and prestige can be considered to be predisposing factors to high SSS.

Dominance and prestige were assessed using the Dominance-Prestige Scale [44]. This is a 17-item, 7-point Likert scale testing dominance (8 items) and prestige (9 items). Internal consistency αs were .88 and .85 for peer-rated dominance and prestige, respectively, and inter-rater αs were .78 and .84, respectively. These high levels of inter-rater agreement suggest that individuals were able to reach a consensus regarding their peers’ dominance and prestige.

Items for dominance include sentences such as “I enjoy having control over others”, whereas items for prestige include sentences as “Members of my peer group respect and admire me”.

#### Moderator

*Personality*. The Big 5 Personality Test [51] was used to provide personality measures. The test contains 50 items, 10 items for each of the five personality traits: extraversion (α > 0.8), agreeableness (α > 0.8), neuroticism (α > 0.7), conscientiousness (α > 0.9), and openness (α > 0.7) where αs are those reported by Alansari [57]. Participants rated themselves as they generally are now, not as they would wish to be in the future. A 5-point Likert scale ranging from 1 (‘Very inaccurate’) to 5 (‘Very accurate’) was used. An example item is “I get stressed out easily”.

### Procedure

This study is part of a broader, repeated cross-sectional investigation into the psychological and social transitions experienced by first-year undergraduate students. During the data collection period, students were invited to participate in a diversity intervention project. This project was not a randomized controlled trial; instead, participation was voluntary to avoid the potential negative effects of compulsory engagement, which might inadvertently reinforce prejudice, the very issue we aimed to mitigate. The intervention was implemented through a departmental diversity forum, allowing students to anonymously post and comment on diversity-related topics.

No significant differences related to the intervention were found in initial multilevel analyses aimed at evaluating the impact of engagement with the diversity intervention between pre- and post-tests. Therefore, the variable representing intervention engagement was included in the initial analyses for this study but was later excluded from the final models due to its lack of significant effects.

The study’s design included three data collection points: at the start of the first semester in October, four weeks into the semester in November, and at the beginning of the second semester in January. To ensure each participant’s data was represented only once in the Structural Equation Modelling (SEM) analysis, we calculated the average scores for each participant across the measured variables. This approach is typically used in data preparation for analysis, particularly in studies involving repeated measures, to simplify the dataset to a per-participant basis for more straightforward statistical analysis, thus maintaining the assumption of data independence. Bakdash and Marusich highlighted that averaging repeated measures can resolve issues of non-independence and ensure data suitability for statistical models that assume independent observations [58]. In the context of executive functioning research, several studies have underscored the importance of this approach when incorporating cognitive measures in SEM analyses [59, 60]. This broad application across diverse research domains underscores the method’s versatility and reliability in handling complex datasets.

After participating in all three sessions, participants were debriefed about the study’s objectives.

### Design

According to the theoretical model (Fig 1), the dependent variable was belongingness. peer status was the mediator. The mental state factors were mental wellbeing and stress. The personality factors were two dimension of the Big-Five personality scale that have been deemed plasticity traits [16], extraversion and openness (to experience).

In a study with six variables, there is an increased likelihood that both direct and indirect effects influence results, as well as the possibility of unobserved or latent variables influencing the relationships. For this reason, we employed a path analysis, followed by a model using structural equation modelling (SEM) to examine the complex interaction between factors. This two-step approach is an established method and has been employed in other studies. For instance, there is a social science study that used SEM to verify the potential path models between perceived racial discrimination and self-rated stress [61]. In another longitudinal study on paranoia by Fowler et al. [62], SEM was initially used to identify paranoia-related latent factors, which were then incorporated into a path analysis to find the most suitable prediction model for paranoia. These examples further validate the chosen combined approach.

Path analysis is a statistical technique that examines direct and indirect relationships among variables [63]. Therefore, we first employed path analysis using Full Information Maximum Likelihood (FIML) estimation in LISREL student version 8.8 [64] to provide a foundation for understanding the direct and indirect effects of mental state and personality on status and belongingness. In this method, missing values are not replaced or imputed, but the missing data is handled within the analysis model. Path analysis enabled us to create a preliminary model that mapped the relationships among variables and to hypothesize the pathways through which they influence one another. Factor whose paths to status and belongingness were insignificant (p > .05) were removed. After a path model was constructed, it was then tested against the specific relationships illustrated in Fig 1.

Once the path model was established, SEM using FIML estimation in LISREL student version 8.8 was conducted to test the mediation and moderation effect. SEM is particularly useful in this context because it allows for the simultaneous analysis of multiple relationships, latent variables, and measurement errors [65]. It also provides model fit indices, which help to assess how well the proposed model explains the data.

While a single equation linear regression model can be used to explore relationships between variables, the former does not offer the same level of flexibility and comprehensiveness as path analysis and SEM, as the latter involves a system of simultaneous regression equations, allowing for more complex relationships, such as mediation and latent factors [65].

## Results

### Descriptive statistics

A total of 209 first-year undergraduates were surveyed. Data collection complied with general data protection regulation (GDPR) guidelines [66]. All data were collected solely by the first author (one person) and were not shared with any other parties. Group means were calculated based on this cross-sectional sample. Descriptive statistics are summarized in Table 2.

**Table 2:** Descriptive statistics of variables.

Variable	M	SD
Dependent variable		
Belongingness	34.48	5.31
Mediator		
Peer status: subjective social status	6.11	1.43
Peer status: dominance	2.94	0.84
Peer status: prestige	4.85	0.77
Independent variable		
Mental state: Mental wellbeing	46.48	8.02
Mental state: Stress	30.55	5.79
Moderator		
Plasticity traits: extraversion	28.80	7.22
Plasticity traits: openness	35.20	5.64

*Note*. N = 209.

### Confirmatory factor analysis of latent variables

The initial phase of our analysis involved performing a confirmatory factor analysis (CFA) using AMOS 28 to evaluate the structure of the latent variables depicted in Fig 1. We incorporated six observed variables into the model to estimate three underlying constructs: mental state (mental wellbeing and stress), social status (subjective social status, dominance, and prestige), and plasticity (extraversion). The model was specified to allow these latent constructs to intercorrelate while preventing any correlations among the unique error terms of the observed variables. The CFA results supported our hypothesized structure, with all observed variables demonstrating significant and meaningful loadings on their corresponding latent factors. The factor loadings varied from moderate to high (λ = 0.25 to 0.97), with an average magnitude of 0.60, suggesting that the indicators reliably represented their respective constructs. The intercorrelations among the latent variables were found to be low to moderate, with values ranging from r = 0.64 (p < .001) to r = 0.83 (p < 0.001), indicating distinct yet related constructs. These significant correlations reinforce the interconnectedness of mental state, plasticity, and peer status. Detailed factor loadings are presented in Table 3.

**Table 3:** Parameter estimates of observed variables (right) to latent variables (left).

Factor loading	Estimate (Standardized)	p-value
Mental state <- Mental wellbeing	0.97	p < .001
Mental state <- Stress (reversed)	0.64	p < .001
Peer status <- Subjective social status	0.67	p < .001
Peer status <- Dominance	0.25	p < .001
Peer status <- Prestige	0.88	p < .001
Plasticity traits <- Extraversion	0.40	p < .001
Plasticity traits <- Openness	0.41	p < .001

Despite a statistically significant Chi-square value, χ2 (11) = 28.55, p = 0.003, the ratio of Chi-square to degrees of freedom, χ2/df = 2.60, fell within an acceptable range [67, 68]. Further, other fit indices confirmed the adequacy of the model: Bentler–Bonett’s normed fit index (NFI) stood at .93, the comparative fit index (CFI) reached .96, and the Akaike Information Criterion (AIC) was 76.55. Collectively, these measures lead us to conclude that the hypothesized latent variable structure was reliably replicated and validated through the CFA process.

### The mediating role of peer status

In our study, we utilized Structural Equation Modelling (SEM) to explore the role of peer status (comprising subjective social status, dominance, and prestige) as a mediator in the relationship between Mental state (comprised of mental wellbeing and stress) and Belongingness. This cross-sectional analysis was conducted using IBM SPSS Amos software v.28.

The primary objective was to evaluate the direct influence of Mental state on Belongingness. Subsequently, we expanded our model to include peer status as a mediator to determine its potential to mediate the effect of Mental state on Belongingness.

To accurately estimate the mediation effect, we employed bootstrapping with 5000 samples. This method is recommended for its effectiveness in generating 95% bias-corrected confidence intervals (CIs) for indirect effects. According to Hayes [69], mediation is considered statistically significant if the 95% CI for the indirect effect does not encompass zero.

In our SEM analysis, we controlled for potential covariates and allowed for correlations among the predictor, mediator, and outcome variables to ensure a comprehensive evaluation of their interrelationships. The model’s goodness of fit was assessed using several indicators: a Chi-square test, the Goodness of Fit Index (GFI) with a value above 0.90 indicating good fit, the Comparative Fit Index (CFI) where values greater than 0.95 suggest excellent fit, and the Root Mean Square Error of Approximation (RMSEA), with values below 0.06 to 0.08 denoting acceptable to good fit. These criteria align with recommendations by Schreiber et al. [70] for evaluating SEM model fit.

**Fig 2:**
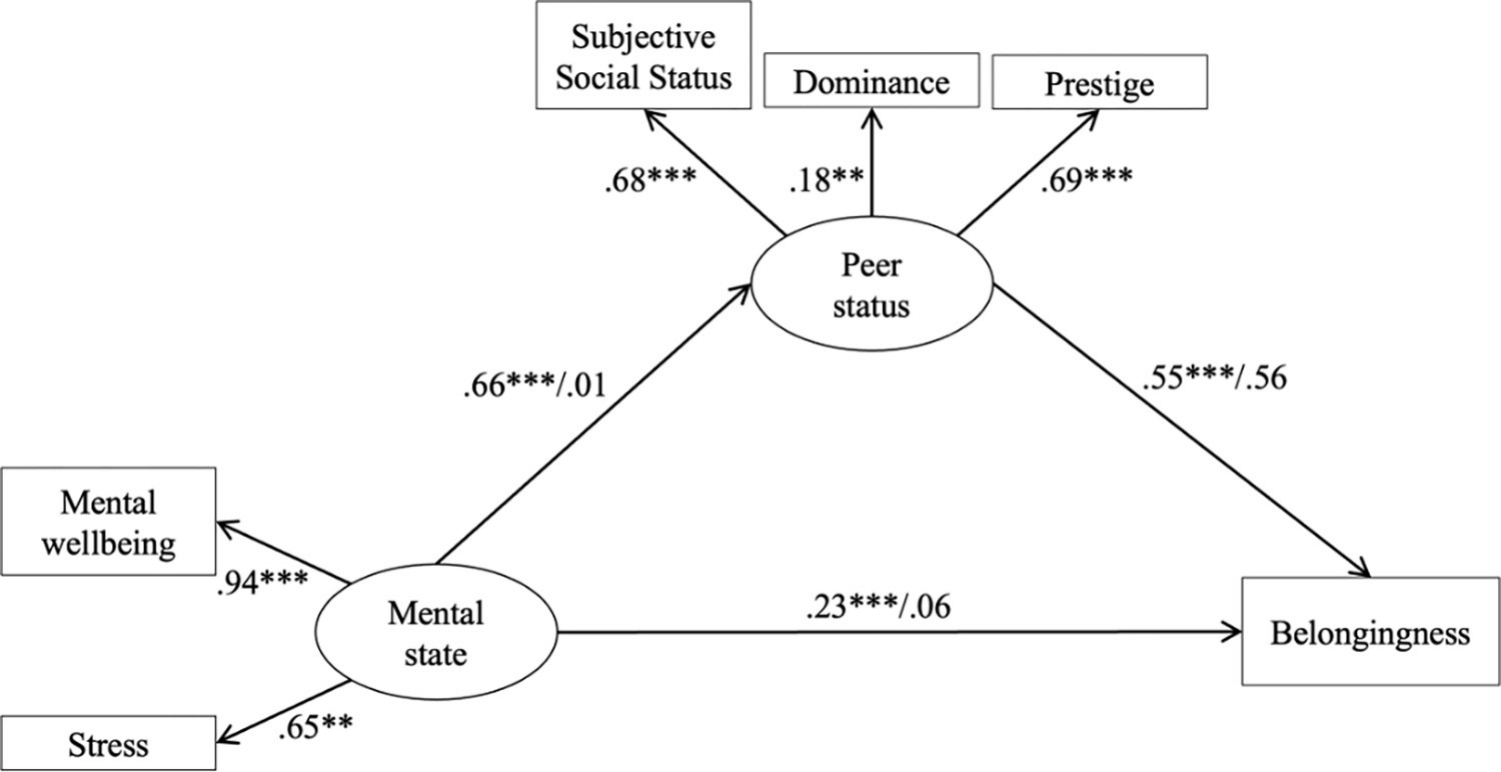
Results of the mediation model (standardized effects). **p < 0.01. ***p < 0.001.

The first SEM model tested the mediator role of status. The results showed that higher mental state predicts higher peer status (β  =  0.66, p < .001). Higher peer status was associated with higher belongingness (β  =  0.55, p < .001). Therefore, the analysis supported the hypothesis, assuming that peer status is a significant mediator between mental state and belongingness. The indirect effects of mental state through peer status on belongingness (β  =  0.23, p < .001, 95% CI [0.16; 0.34]) was significant. Thus, peer status fully mediated the hypothesized relationships. The observed variable estimates were also significant (see Fig 2). The Chi-square statistic was acceptable given the large sample: χ^2^(13) = 27.38, p = .01. Indicators of the fit for the model indicated an excellent fit: Goodness of Fit Index (GFI) = .98, comparative fit index (CFI) = .99, root mean squared error of approximation (RMSEA) = .07.

### The moderation role of plasticity traits

**Fig 3:**
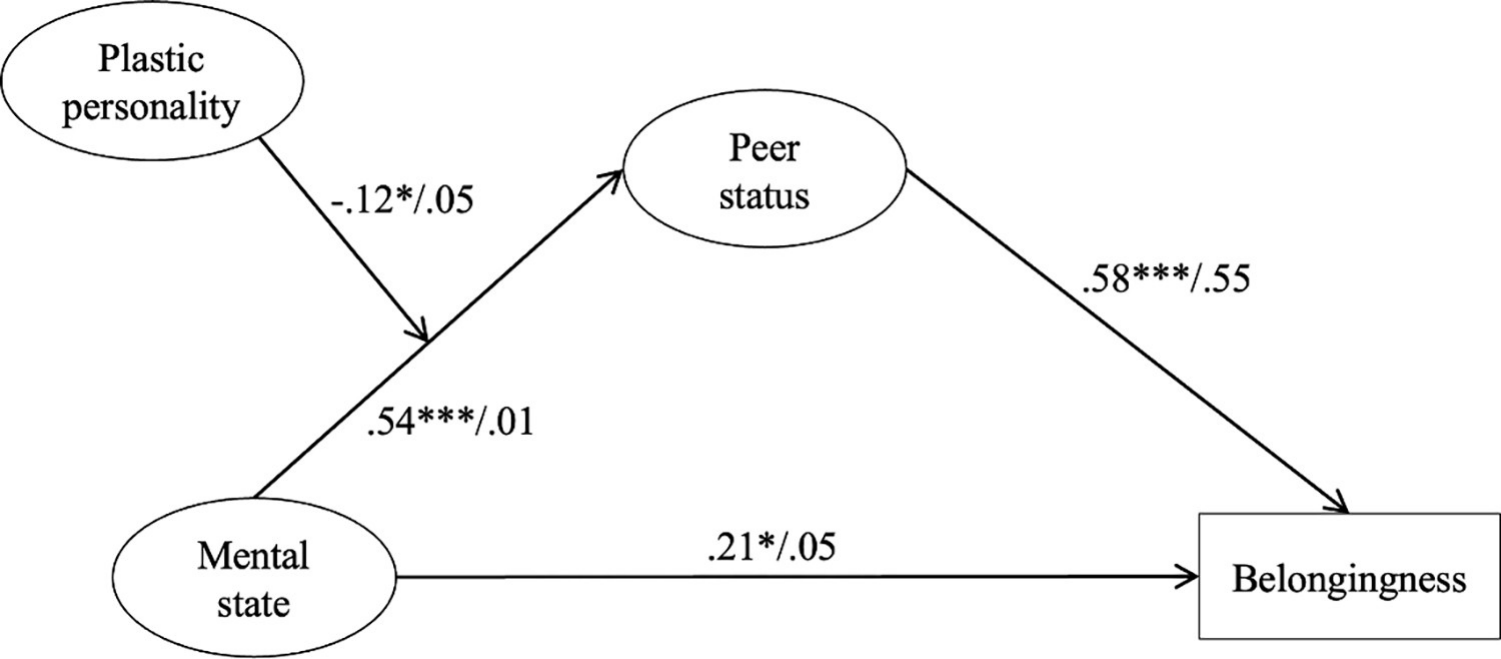
Results of the moderation model (standardized effects). *p < .05. **p < .01. ***p < .001.

The next model (Fig 3) was employed to examine the moderating role of plasticity traits (extraversion and openness) on the relationship between mental state and peer status. To address potential issues of multicollinearity and facilitate the interpretation of the interaction term, both mental state and plasticity were standardized to obtain their respective z-scores. The interaction term was then created following the procedure introduced in Cortina et al. [71] by multiplying these z-scores, thus representing the combined effect of mental state and plasticity traits on social status in a standardized metric. The standardization process prior to the creation of the interaction term effectively reduces multicollinearity, ensuring more reliable estimates of the regression coefficients. Plasticity alone significantly predicted peer status (β  =  .38, p < .001, 95% CI [0.16; 0.34]). Furthermore, the interaction between the z-scores of mental state and plasticity on peer status was significant (β  =  -.12, p = .03, 95% CI [-0.22; -0.01]). While the hypothesis expected that higher plasticity would amplify the positive effect of mental state on peer status, the findings suggest the opposite. The model demonstrated an adequate fit to the data: Chi-square χ2(21) = 58.68, p < .001, with Goodness of Fit Index (GFI) = .95, Comparative Fit Index (CFI) = .94, and Root Mean Squared Error of Approximation (RMSEA) = .09. These indices collectively suggest a satisfactory fit of the model.

## Discussion

This study used a social ecological model to examine the predictive ability of mental state on the belongingness of first-year undergraduate students. We also examined the mediating role of peer status on mental state and belongingness, and the moderating effect of personality traits on mental state and peer status. The findings provided strong evidence for the positive effect of mental state on undergraduate belongingness, how students’ subjective popularity (peer status) could mediate the effective of mental state, and how plastic traits like extraversion and openness to experience could strengthen the impact of mental state of social status.

The first hypothesis stated that mental state should be positively associated with university belonging. The final SEM model (Fig 1) showed that mental state explained 21% of the variability in belongingness. This supported the idea that the influence of personal attributes on the belongingness in educational settings is significant. Moreover, the findings indicated that such impact is not confined to secondary education but also persists in university environments [36, 39, 72, 73]. These results underscore the importance of mental health initiatives in universities, which could include the early screening of mental health difficulties for first year undergraduates by the school’s wellbeing team. Research demonstrated that for youths from lower socio-economic backgrounds, social support can serve as a crucial protective factor for them, potentially mitigating the effects of depressive symptoms [17, 74]. Therefore, university intervention strategy could include peer and staff support, possibly in the form of online peer-support forum, mental health counselling, or wellbeing workshops.

For the second hypothesis, we discovered that higher mental state leads to higher status among peers, which subsequently enhance first-year students’ belongingness. In agreement with a social ecological model, this finding highlighted the interaction of individual level and micro-level factors on university belongingness. Previous meta-analysis on secondary school belongingness suggested that peer relationship had a smaller contribution to belongingness than parent and staff support, this the current findings may suggest a different dynamic in university setting [75]. This finding might not be so surprising as young adults move out from parental home to attend higher education. This transition often leads them to rely more on their peers as figures of attachment [76]. A growing literature highlights the crucial role of peer relationship in university transition, where the most significant determinant of university belongingness is identified as high quality of peer attachment [76–79]. Undergraduates who established strong connection among peers, more university friends, and higher friendship quality reported higher attachment to university life as well as to the university. On the other hand, positive individual traits, such as mental state could facilitate the establishment of peer relationships, as students with a stable and secure identity are more approachable and trustworthy, and they also have the capacity to maintain a long-term friendship [44]. Consequently, universities could foster the development of peer relationships among students through institution-endorsed initiatives such as mentorship programs and orientation events at the beginning of the academic term. University administrators may motivate faculty and staff to facilitate environments conducive to social interaction, ensuring they are accessible for both personal and academic advice. Additionally, directing students to appropriate peer support groups or counselling services should be advocated when they encounter challenges that necessitate specialized assistance.

Finally, the moderating effect of plasticity traits proposed in the third hypothesis was partially supported. Plasticity independently predicts higher peer status. However, contrary to the Hypothesis 3, higher plasticity reduces the positive impact of mental state on peer status. With respect to the recent recognition of student personality of their overall wellbeing [21, 80], the predictability of plasticity to peer status may verify the behavioural markers of plastic traits: leadership, skilfulness, and expressiveness in social situations [81]. Extraversion, for instance, predicts university satisfaction at senior years and positive social experiences throughout undergraduate, with more extraverted students were more satisfied with college and had more positive social experiences [41]. Nonetheless, the extent to which plasticity interacts with a positive mental state to achieve status is unclear. The Unifying Theory of Personality can support the negative moderation effect of plasticity. The theory states that plasticity traits primarily manifest as a sensitivity to rewards, encompassing not just social rewards but also non-social stimuli like physical activity and positive emotions [16, 33, 82, 83]. Extraversion for instance, often considered a social trait, indeed enhances sensitivity to social rewards such as status and affiliation [33]. However, its broader spectrum includes a general propensity towards reward-seeking behaviours, explaining why individuals high in extraversion (sensitivity to specific rewards) and openness (sensitivity to the reward value of information) may pursue a variety of experiences–including those related to food, warmth, sex, and affiliation–that do not directly contribute to social status. A longitudinal study tracking high school students’ transition to higher or vocational education revealed that although plasticity traits predict more positive social experiences and interpersonal relationships, individuals who are more extraverted and open were also more reluctant to psychotherapy and more likely to engage in activities such as sleeping, eating, sex, and finances [79]. Thus, a better mental state might not always lead individuals with higher plasticity traits to seek social status. Instead, positive mental state might serve as motivational energizer that allow individuals to seek a wider array of rewards, both beneficial and potentially risky. This underscores the complex role that plasticity traits play in personality development and their influence on social dynamics. Certain traits, such as kindness, may exert a greater influence or may be more amenable to intervention than others [84, 85]. There is a need for additional research to identify which traits are crucial and the most effective methods for developing them. Future research should focus on exploring how plasticity traits interact with levels of mental states across different contexts, such as academic environments, extracurricular activities, and personal relationships. It would be beneficial to conduct more longitudinal studies to examine the changes and impacts of plasticity traits over the entire university period. Additionally, investigating the efficacy of targeted interventions that aim to enhance or moderate specific aspects of plasticity traits could provide valuable insights. Such interventions could focus on promoting young adults’ adaptive expressions of traits like extraversion and openness while mitigating potential risky behaviours.

Some limitations might limit the validity of the results. Although this study focused individual level predictors of university belongingness, demographic factors were not collected and could not be included in the model. Take the individual traits, ethnicity, as an example. British students from ethnic minority backgrounds are less likely to be admitted to prestige universities than their comparably qualified white British peers [86] and are also less likely to be awarded a first or upper second-class degree after entry to university [87]. Therefore, a diminished belongingness to the university among ethnic minority students could be predetermined by their rejection from their preferred universities and exacerbated by unsatisfactory grades at the end of their first year. These two critical time points were not examined in this study. Hence, future research should undertake a longitudinal study of an undergraduate cohort from entry to graduation to observe potential shifts in the social status and belongingness. Additionally, qualitative studies should explore the transition experiences of students with specific characteristics (e.g., introverted, ethnic minority) for a more nuanced understanding.

Secondly, our study utilized a cross-sectional study adapted from a larger repeated cross-sectional design. This was due to that we encountered challenges with participant retention, as only one-third of participants completed all three data collection sessions. This low retention rate may have obscured the potential effects of the intervention [80]. The primary reason for this dropout was the study’s voluntary nature and seasonal assessment timetables. We chose not to mandate participation to avoid the unintended consequences of enforced engagement, which, paradoxically, could exacerbate prejudice—the exact opposite of our goals [81]. Opting for voluntary participation, despite the risk of lower retention, seemed preferable to compromising the integrity of the study’s objectives.

Additionally, the online distribution of both the questionnaire and the intervention contributed to our challenges with participant engagement, a known issue with online formats that lack compelling engagement (“stickiness”) [80, 82, 83]. The anonymity and inability to trace responses further complicated retention. Future studies might overcome these issues by implementing strategies such as using shorter questionnaires, offering electronic cash incentives, or sending reminders to non-respondents [80]. A meta-analysis suggests that minimizing participant burden through various data collection methods could be the most effective retention strategy [84], providing a roadmap for enhancing engagement in online longitudinal studies while preserving anonymity.

Alternatively, future research could consider a true cross-sectional design. Despite the preference for longitudinal studies in examining university transition [7], cross-sectional research could circumvent the challenges of participant variability over time. Such an approach would eliminate the influence of time and experience on university belongingness, which tends to increase as students advance through their university years [85]. Utilizing the conceptual framework established in our current study, cross-sectional research could offer fresh insights by focusing different university year groups on specific points in time, reducing the complexities associated with tracking changes over extended periods.

In conclusion, while belongingness has been repeatedly shown to be significant factors for successful transition to university and subsequent success, the present study explored the structural component of first-year undergraduate’s belongingness. We established a model for the status and belongingness of first-year undergraduates by examining the direct effects of mental state and the indirect effects of individual traits. We investigated the interaction of multiple factors and the effectiveness of diversity intervention on first-year undergraduates attending UK universities. The key takeaway from our research is the significant role that peer status plays in mediating mental state and students’ belongingness. Traditionally, social class has been seen as a static factor influencing student belongingness. It suggests that university students from lower socio-economic backgrounds are typically at a disadvantage in feeling a belongingness, except in cases where these students have had access to elite education before attending university [88, 89]. However, our study introduces a fresh angle by focusing on peer status–a more malleable aspect of social class. This provides a new pathway for universities to support students from lower socio-economic backgrounds. By offering opportunities for these students to elevate their status among peers–such as through leadership roles specifically for marginalized students or involving them in co-designing programs—universities can enhance their belongingness. This approach challenges the predetermined notion of a mismatch for lower-class students, suggesting that enhancing peer status peers can help break the cycle of disadvantage.
